# Pathogenic *Neisseria* Hitchhike on the Uropod of Human Neutrophils

**DOI:** 10.1371/journal.pone.0024353

**Published:** 2011-09-16

**Authors:** Niklas Söderholm, Katarina Vielfort, Kjell Hultenby, Helena Aro

**Affiliations:** 1 Department of Genetics, Microbiology and Toxicology, Stockholm University, Stockholm, Sweden; 2 Department of Laboratory Medicine, Karolinska Institute, Huddinge, Sweden; Indian Institute of Science, India

## Abstract

Polymorphonuclear neutrophils (PMNs) are important components of the human innate immune system and are rapidly recruited at the site of bacterial infection. Despite the effective phagocytic activity of PMNs, *Neisseria gonorrhoeae* infections are characterized by high survival within PMNs. We reveal a novel type IV pilus-mediated adherence of pathogenic *Neisseria* to the uropod (the rear) of polarized PMNs. The direct pilus-uropod interaction was visualized by scanning electron microscopy and total internal reflection fluorescence (TIRF) microscopy. We showed that *N. meningitidis* adhesion to the PMN uropod depended on both pilus-associated proteins PilC1 and PilC2, while *N. gonorrhoeae* adhesion did not. Bacterial adhesion elicited accumulation of the complement regulator CD46, but not I-domain-containing integrins, beneath the adherent bacterial microcolony. Electrographs and live-cell imaging of PMNs suggested that bacterial adherence to the uropod is followed by internalization into PMNs via the uropod. We also present data showing that pathogenic *Neisseria* can hitchhike on PMNs to hide from their phagocytic activity as well as to facilitate the spread of the pathogen through the epithelial cell layer.

## Introduction

The genus *Neisseria* includes the two obligate human pathogens *N. gonorrhoeae* and *N. meningitidis. N. gonorrhoeae* primarily colonizes the urogenital mucosa, where it crosses the intact mucosal barrier and remains in the tissue to initiate an inflammatory response [1]. *N. meningitidis* is commonly found in the nasopharynx of healthy individuals, where it can cross the mucosal epithelium and cause sepsis and/or meningitis. Bacterial dissemination across the blood brain barrier (BBB) has been reported to occur by hijacking the β-adrenoreceptor/β-arrestin pathway, triggering the opening of the intercellular junctions of the brain-endothelial interface [2].

Bacteria can also enter the meninges from the nasopharynx by redistribution of the intracellular junction protein N-cadherin in olfactory epithelia [3]. Although these two species of bacteria cause different diseases, the molecular mechanisms during infection are similar and they share many important virulence factors involved in both adhesion to and invasion of epithelial cells. Initial adherence by *Neisseria* to host epithelial cells is mediated by type IV pili (T4P) [4], [5], which elicit cortical plaque formation in epithelial cells [6]. It has been reported that T4P of pathogenic *Neisseria* bind two different types of receptors: the complement regulator CD46 [7], [8] and the I-domain-containing integrins on epithelial cells [9]. However, the role of CD46 in adhesion of *Neisseria* is yet not fully elucidated [10]. Upon infection, CD46 is phosphorylated at tyrosine 354, clusters beneath microcolonies of *N. gonorrhoeae* and induces a transient release of Ca^2+^ from intracellular stores [11], [12], [13]. Bacterial adhesion to epithelial cells depends on the major pilus subunit protein PilE [14], [15] and the pilus associated protein PilC [16], [17], [18], [19]. PilC undergoes intra-strain phase variation [17] and has been suggested to be the tip-adhesin of the pilus fiber [20]. In *N. gonorrhoeae,* both PilC1 (*pilC2*Δ)- and PilC2 (*pilC1*Δ)-expressing bacteria adhere to epithelial cells, while only PilC1 (*pilC2*Δ)-expressing *N. meningitidis* mediate adherence [21], [22], [23]. In contrast, Morand *et al.* reported that meningococcal PilC2 mediated adhesion to epithelial cells [19].

Initial adherence is followed by tight adherence where the Opacity proteins (Opa) in the bacterial outer membrane function as secondary bacterial ligands to host cell epithelium. Opa interacts with carcinoembryonic antigen-related cell adhesion molecules (CEACAM) and heparan sulfate proteoglycans (HSPG) on epithelial cells [24], [25], [26]. Infection by pathogenic *Neisseria* triggers release of pro-inflammatory chemokines that recruit polymorphonuclear leukocytes (PMNs or neutrophils) to the site of infection [27], [28], [29]. Although PMNs can engulf and kill most microorganisms, *N. gonorrhoeae* is remarkably resistant to PMN killing [26], [30]. In fact, gonorrhea is characterized by a purulent urethral or cervical discharge consisting primarily of PMNs containing intact intracellular bacteria. *In vitro* assays show that PMNs only eradicate up to 70% of piliated *N. gonorrhoeae* added within the first hour [31]. *N. gonorrhoeae* has developed a number of strategies to evade the antimicrobial activity of PMNs, such as IgA protease-mediated degradation of lysosomal LAMP1 [32] and inhibition of signaling pathways required for inducing oxidative burst [26].

Severe meningococcal disease is characterized by early PMN activation. An accumulation of PMNs in the subarachnoidal space is one of the hallmarks of *N. meningitidis* infection [33]. Early neutropenia is associated with a high risk of developing severe meningococcal disease [34], [35]. PMN activation enhances microbial clearance but also contributes to the vascular damage and multiorgan failure associated with meningococcal sepsis. *N. meningitidis* causes a moderate increase in CD11b (Integrin α M β2/complement receptor 3 α subunit) and CD18 (Integrin β2/CR3 β subunit) in PMNs [36]. CR3 participates in pilus-mediated adherence to cervical epithelia [37].

Circulating PMNs isolated from blood are round and non-polarized. PMNs become polarized upon chemotactic activation and contact with a matrix such as epithelial cells or coated glass. The key element that drives cell motility and cytoplasmic remodeling is the activity of the cytomatrix [38]. This physiology of a polarized PMN can be divided into pseudopod, cell body and uropod. The pseudopod at the front of a migrating PMN mediates the phagocytosis of microorganisms. Hence, the uropod is the rear of a moving PMN and possesses no phagocytic activity. Uropods are enriched in adhesion- and signaling-receptors such as integrins, HPSGs, inter-cellular adhesion molecules (ICAMs), actin and ezrin/radixin/moesin (ERMs) [39]. PMNs also express CD46, but at 10-fold lower concentrations compared to epithelial cells [40].

Over 30 years ago, Densen and Mandell observed that single piliated gonococci adhered to the surface of PMNs while non-piliated bacteria were more easily engulfed [41]. Here, we reveal new data showing that pathogenic *Neisseria* adheres by its T4P to the uropod of human PMNs. Meningococcal adherence depends on both PilC1 and PilC2 for adhesion, while *N. gonorrhoeae* adherence does not. Using live cell imaging techniques, we demonstrate a new mechanism where pathogenic *Neisseria* can hitchhike on the uropod of PMNs and thereby avoid the phagocytic activity of the PMNs and simultaneously increase their ability to spread at the epithelial cell layer.

## Results

### N. gonorrhoeae and N. meningitidis adhere to human PMNs

**Figure 1:**
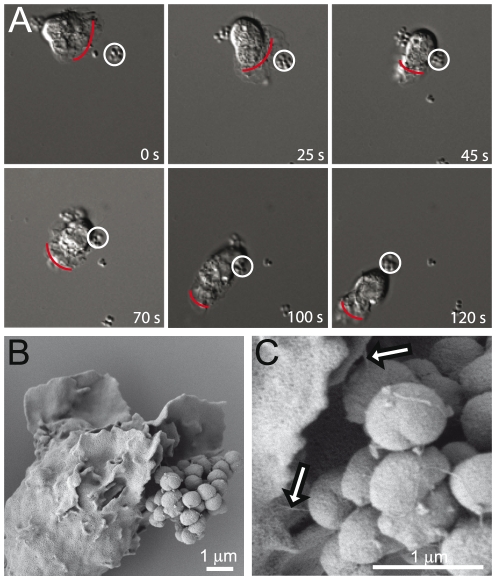
*Neisseria* adhere to human PMNs. (**A**) Image sequences showing the interaction between a bacterial microcolony of *N. gonorrhoeae* and a PMN. The bacterial microcolony escapes engulfment and move along the plasma membrane to the uropod. Images show 120 seconds of live-cell imaging. Red lines indicate the pseudopods and the front of the migrating PMN. The white ring indicates the microcolony of interest. (**B**) SEM electrograph showing pilus-mediated adherence of *N. meningitidis* FAM20 to the plasma membrane on the right side of the pseudopod. (**C**) Magnified image of (B). Arrows indicate pili.

We analyzed the ability of *Neisseria* to adhere to human PMNs. Freshly isolated PMNs were first allowed to adhere to the glass bottom of cell culture dishes. The uropod was distinguished from the pseudopod by the fact that the uropod is at the rear of the migrating PMN. Bacteria were then added to the polarized PMNs at a multiplicity of infection (m.o.i.) of 100. Within 30 minutes of incubation, both *N. gonorrhoeae* MS11 and *N. meningitidis* FAM20 adhered to the uropod of viable PMNs. We followed the interaction by live-cell microscopy for 3 hours during which images were captured every 5 seconds. Bacterial adherence was reversible and whole bacterial microcolonies or parts of a microcolony could disassociate from the uropod. Gonococcal and meningococcal adherence to the uropod occurred either by direct uropod contact or by transport on the plasma membrane from the pseudopod to the uropod. Figure 1A shows a representative image sequence of a microcolony first encountering the pseudopod but evading phagocytosis by migrating on the plasma membrane to the uropod (Fig. 1A, Movie S1). Furthermore, this adherence to the plasma membrane was pilus-mediated, as determined by scanning electron microscopy (SEM) (Fig. 1B and C).

**Figure 2:**
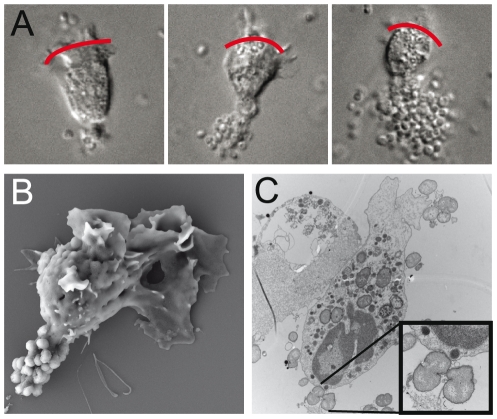
Bacterial microcolonies adhere to the uropod. (**A**) DIC images of viable PMNs with *N. meningitidis* FAM20 adhered to the uropod. Three images show; low bacterial adherence (left), a medium-sized microcolony (middle) and a big microcolony adhering to the uropod (right). Red lines indicate the pseudopods and the front of the migrating PMN. (**B**) SEM electrograph showing the specificity of FAM20 adherence to the uropod of a polarized PMN. (**C**) TEM image showing the interaction between *N. gonorrhoeae* MS11 P^+^ and a PMN. Bacterial uropod adherence is enlarged in the box to the right.

Microcolonies of different sizes and single bacterium could adhere to the uropod of polarized PMNs but microcolonies consisting of approximately ten to one hundred bacteria were more frequently observed. The adherences of a single meningococcus, a small microcolony and a big microcolony are shown in Figure 2A. Identical adherence patterns were seen for gonococci. We confirmed the bacterial-uropod interaction by SEM and transmission electron microscopy (TEM) (Fig. 2B and C, respectively). In Figure 2C, uropod adherence is captured at a later stage when there is a tighter interaction between the bacteria and the PMN prior to internalization (close up). Other bacteria were also captured prior to phagocytosis by the pseudopod protrusions.

Adherence of MS11 and FAM20 to the uropod was also observed in differentiated HL60 cells (Fig. S1), a promyeoloblast cell line that differentiates into neutrophil-like cells after chemical treatment and exhibits phagocytic activity and responsiveness to chemotactic stimuli (Methods S1).

Taken together, these data show that piliated translucent colonies of *N. gonorrhoeae* as well as piliated Opa-positive *N. meningitidis* adhere to the uropod of human PMNs. Bacteria that encounter other sites of the PMN plasma membrane also use their pili to promote adhesion.

### Bacterial adherence occurs frequently and does not impair PMN velocity

**Figure 3:**
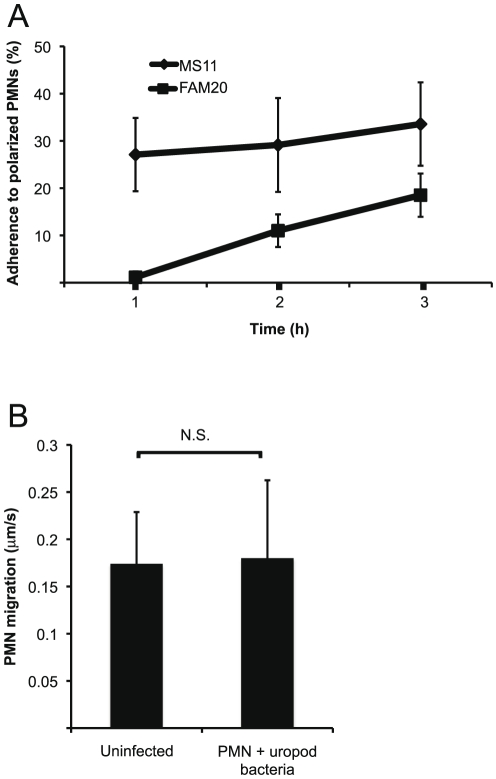
Bacterial adherence occurs frequently and does not impair PMN velocity. (**A**) The frequency of bacterial adherence to a PMN uropod. Graph showing the number of PMNs with bacteria adhering to the uropod after 1 to 3 hours of incubation. The adherence of MS11 and FAM20 to the uropod of PMNs was calculated from more than 300 observed cells in three independent experiments. The average percentages of adhering *N. gonorrhoeae* MS11 and *N. meningitidis* FAM20 and standard deviations are shown. (**B**) Graph showing the average velocity of PMNs migrating on glass. Differences in average velocity were determined by Student *t*-test (p<0.05). The mean velocity of uninfected and infected PMNs and standard deviations are shown.

Next, we quantified the frequency of uropod-specific bacterial adherence. The interactions between PMNs and bacteria were monitored during 1 to 3 hours of incubation by live-cell imaging and at least 100 cells were monitored. At each time point, images of randomly selected microscopic fields were captured and the PMNs were counted. No differences were found between PMNs carrying single bacterium or microcolonies when quantifying the frequency of uropod adherence. However, PMNs carrying the amount seen in Figure 2A (middle) was the most common. Already after 1 hour, *N. gonorrhoeae* MS11 bound more frequently to the uropod than *N. meningitidis* FAM20. The number of PMNs with adhering bacteria on the uropod increased during the incubation and after 3 hours, 16% of the PMNs observed had bound *N. meningitidis* FAM20 and 33% had bound *N. gonorrhoeae* MS11 (Fig. 3A). The experiment was repeated three times with PMNs isolated from different blood donors. The frequency of adherence varied slightly between the blood donors. The number of bacteria adhering to the uropod varied with the size of the microcolony (as shown in Fig 2A) and was dynamic all through the analysis. Hence, the number of adhering bacteria per PMN was not calculated.

We investigated whether the burden of a big bacterial microcolony on the uropod impaired PMN motility. The velocity of a PMN carrying a big microcolony on the uropod was measured in live-cell conditions using a tracking module. The motility of the PMN was not affected since both PMNs alone and PMNs with adhering bacteria moved on glass with a velocity of about 0.15 to 0.20 µm/s (Fig. 3B).

### The pilus promotes bacterial adherence to the uropod and depends on PilC for binding

**Figure 4:**
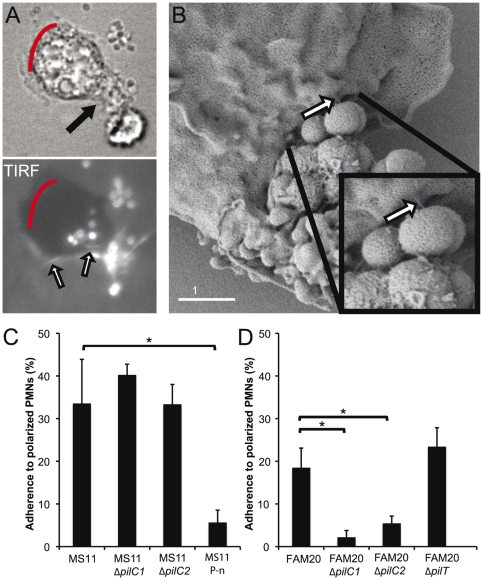
Pili promote adherence of *Neisseria* to the uropod and meningococcal adherence depends on PilC expression. (**A**) The interaction between DyLight 488 NHS ester-stained pili and the PMNs was observed under the microscope in TIRF using a connected argon laser and a 100x objective (N/A 1.46). Red lines indicate the pseudopod. Upper panel: the PMN in bright field image. Arrow indicates one adhering microcolony of FAM20 bacteria. Lower panel: TIRF image of the PMN. White arrows indicate two pili bound to the plasma membrane of the uropod. (**B**) SEM electrograph showing the direct pilus-uropod interaction. (**C**) Graph showing the average number of PMNs with *N. gonorrhoeae* MS11 and mutants adhering to the uropod. (**D**) Graph showing the average number of PMNs with *N. meningitidis* FAM20 and mutants adhering to the uropod. Bacterial adherence was calculated from more than 300 observed cells in three independent experiments. One hundred cells were observed with the FAM20 Δ*pilT*. Statistical significance was calculated in C and D by one-way ANOVA. Data were considered significant if *P*<0.05 and indicated as *.

Since pilus interactions with host cells play a major role in bacterial adherence to epithelial cells, we also determined the role of T4P in uropod adherence to human PMNs. To visualize T4P in living cells, piliated bacteria were labeled with a fluorescent DyLight 488 NHS ester and imaged by Total Internal Reflection Fluorescence (TIRF). With this technique, the total reflection of a laser generates an evanescent field that yields high resolution within a few nanometers above the object glass. Hence, the fluorescently labeled pili become visible [42]. Single bundles of fluorescent T4P could be detected at the uropod in TIRF within 60 minutes of bacterial incubation (Fig 4A, Movie S2). This was never observed in non-piliated gonococcal or meningococcal strains (data not shown). These data clearly showed that pili extending from the microcolonies directly mediated binding between the bacteria and the uropod of viable PMNs. The pilus binding to the uropod was confirmed in SEM electrographs (Fig. 4B). Thus, T4P mediate binding between the bacteria and the uropod of a PMN.

We then determined the importance of piliation, PilC and PilT in adherence to uropod. *N. gonorrhoeae* and *N. meningitidis* wild-type strains and mutants were incubated with freshly isolated human PMNs at m.o.i. 100 for 3 hours. As stated previously, the piliated *N. gonorrhoeae* strain MS11 P^+^ adhered to 33% of the cells observed, while the non-piliated strain MS11 P-n (in which the 5′ end of *pilE* was deleted) adhered to 5% of the cells observed (Fig. 4C). Neither the gonococcal PilC2 deficient mutant MS11 *pilC2^−^* (P^+^, *pilC2*::mTnCm) nor the PilC1 deficient mutant MS11 *pilC1^−^* (P^+^, *pilC1*::mTnCm) was significantly different from that of MS11 wild-type, binding to 32% and 40% of PMNs, respectively (Fig. 4C). However, the MS11 PilC double knockout (*pilC1*::mTnCm, *pilC2*::mTnCm) mutant resulted in a non-piliated phenotype [17] and behaved like the non-piliated MS11 P-n (data not shown). On the contrary, both meningococcal PilC1 deficient mutant FAM20 Δ*pilC1* (P^+^, PilC2^+^, *pilC1*::mTnCm) and the PilC2 deficient mutant FAM20 Δ*pilC2* (P^+^, PilC1^+^, *pilC2*::mTnCm) showed reduced uropod adherence and adhered after 3 hours to only 2% and 5% of PMNs, respectively (Fig. 4D). In addition, the non-piliated strain JB515 P*^−^* (serogroup W135) [43] adhered to less than 5% of the PMNs counted (data not shown), as seen also for the non-piliated MS11 strain. The hyper-piliated PilT mutant (FAM20Δ*pilT*) was not affected in its ability to bind to the uropod (Fig 4D).

Taken together, these data show that T4P mediate bacterial adherence to the uropod of human PMNs while non-piliated *Neisseria* interacts very infrequently with PMNs. Meningococcal uropod adherence depends on both PilC1 and PilC2, while gonococcal uropod adherence is not affected by single knock outs of PilC1 or PilC2.

### Uropod adherence is specific for *Neisseria* species

Non-pathogenic commensal *Neisseria* species were also tested for PMN adherence. *Neisseria subflava* and *Neisseria lactamica* both exhibited adherence comparable with that of MS11 P-n, *i.e.*, to less than 5% of the PMNs (Table 1). However, occasional pili-like structures were detected on these two strains by TIRF (Fig. S2, Methods S1) but none of these non-pathogenic strains express PilC [17]. Moreover, *Streptococcus pyogenes*, *Escherichia coli* and the type IV pili-expressing *Pseudomonas aeruginosa* did not adhere to the PMNs (Table 1).

**Table 1:** The uropod adherence is specific for *Neisseria* species.

Strain	PMN uropod adherence
*Neisseria subflava*, non-pathogenic	<5%
*Neisseria lactamica*, non-pathogenic	<5%
*Streptococcus pyogenes*, S165, T6, emm6	none
*Escherichia coli,* DH5α	none
*Pseudomonas aeruginosa*, PA32	none

Next, we analyzed the importance of Opa proteins in bacterial uropod adherence. Several morphologically different Opa-positive colonies were selected from *N. gonorrhoeae* MS11 under a binocular microscope. Opa-positive bacteria were incubated with PMNs as described previously. No alterations in adherence were seen for different MS11 P^+^, Opa^+^ bacteria compared to MS11 P^+^, Opa^-^ bacteria (data not shown). However, these results do not include some Opa-expressing bacteria that do not increase colony opacity. We therefore examined the adherence of translucent MS11 P-n colonies, which would include both Opa-negative and translucent Opa-positive bacteria. Although extensively repeated, no difference in adherence was observed. The role of these bacteria will be of future interest. We also tested whether bacteria or PMNs have to be viable for bacterial adherence. Indeed, no uropod-specific adherence could be detected after 3 hours of incubation when PMNs or bacteria were fixed with paraformaldehyde prior to infection (data not shown).

### CD46 accumulates at the uropod upon bacterial adherence

**Figure 5:**
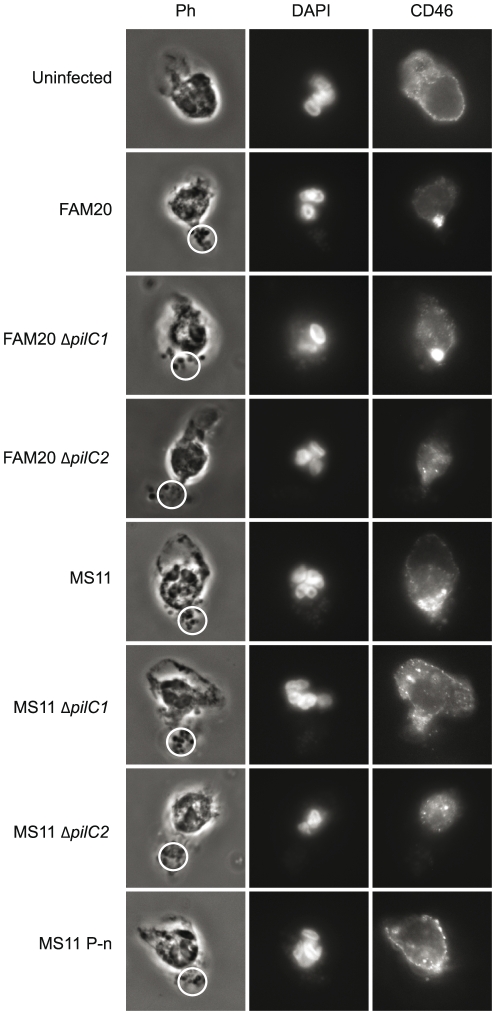
CD46 accumulates at the uropod adjacent to adhering bacteria. *N. gonorrhoeae* MS11 and *N. meningitidis* FAM20 bacteria were allowed to adhere to freshly isolated PMNs. Cells were fixed, permeabilized and incubated with polyclonal antibodies against CD46 and cellular DNA was stained with DAPI. Phase contrast images show the PMN morphology and bacterial adherence. Circles indicate bacteria. Representative images of CD46 expression in PMNs infected with wild-type and mutant gonococci and meningococci are shown.

We hypothesized that a cellular component important for uropod-specific adherence might be enriched at the uropod. Consequently, we first analyzed the role of CD46 as a pilus receptor of PMNs. CD46 was blocked with polyclonal antibodies against CD46 before infection but no inhibition of bacterial adherence was detected (data not shown). However, immunofluorescence staining with antibodies revealed that CD46 had accumulated at the uropod adjacent to the adhering bacteria (Fig. 5). Adherence of FAM20 P^+^ and the PilC2-expressing mutant FAM20 Δ*pilC1* induced a redistribution of CD46 to the uropod, while that of FAM20 Δ*pilC2* did not (Fig. 5). Hence, the recruitment of CD46 at the uropod depends on PilC2. Uninfected PMNs and PMNs incubated with the non-piliated MS11 P^-^n showed equally distributed CD46 at the cell surface (Fig. 5).

**Figure 6:**
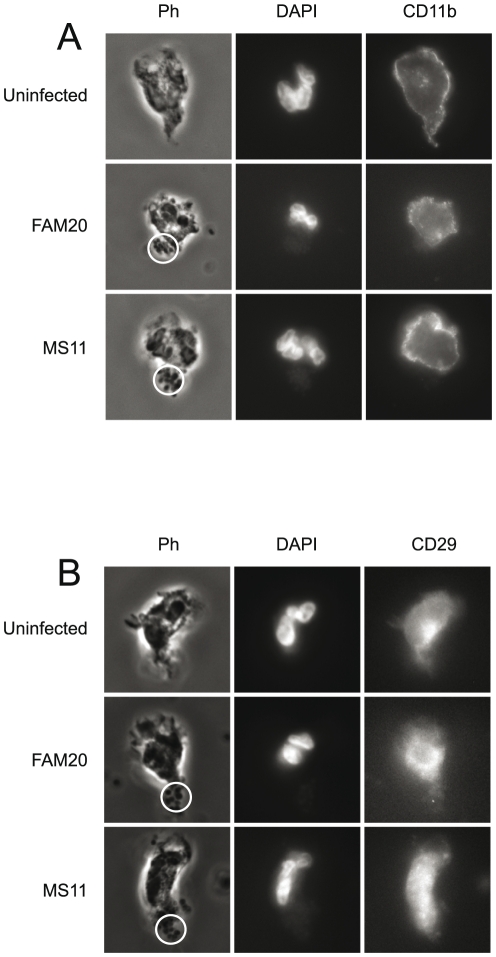
CD11b and CD29 expression in infected and uninfected PMNs. *N. gonorrhoeae* MS11 and *N. meningitidis* FAM20 bacteria were allowed to adhere to freshly isolated PMNs. Cells were fixed, permeabilized and incubated with monoclonal antibodies against CD11b or CD29 and cellular DNA was stained with DAPI. Phase contrast images show the PMN morphology and bacterial adherence. Circles indicate bacteria. (**A**) Representative images of CD11b expression in PMNs infected with wild-type and mutant gonococci and meningococci. (**B**) Representative images of CD29 expression in PMNs infected with wild-type and mutant gonococci and meningococci.

I-domain-containing integrins function as pili receptors important for gonococcal adherence to epithelial cells [9]. Therefore, we also investigated the role of both CD11b (integrin α1) and CD29 (integrin β1) in bacterial-uropod interaction. Pre-incubation with antibodies against either CD11b or CD29 did not reduce bacterial adherence (data not shown). Also, there was no accumulation of CD11b or CD29 at the uropod (Fig. 6A and B).

Taken together, these data show that CD46 is recruited beneath the site of meningococcal adhesion and is PilC2-dependent. During gonococcal adherence, both PilC1 and PilC2 expression is required for successful CD46 redistribution.

### Intracellular bacteria at the uropod indicate bacterial internalization

**Figure 7:**
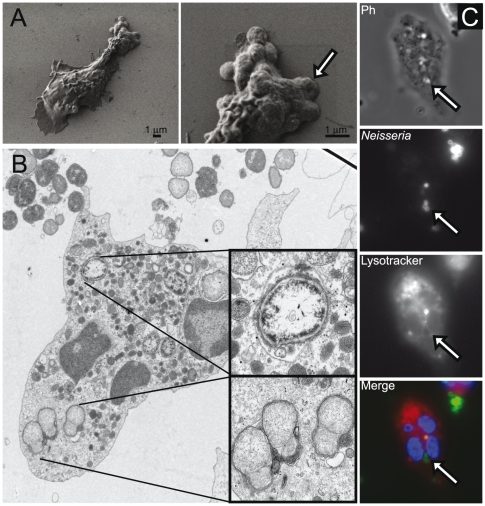
Bacteria invade PMNs via the uropod and intracellular bacteria can be found close to the uropod. (**A**) *N meningitidis* FAM20 were allowed to adhere to the uropod of freshly isolated PMNs. Left electron micrograph shows intracellular bacteria at the uropod. Right electron micrograph shows a magnified image of the cellular uropod at 16 000 x magnification of the same PMN. Arrow indicates an intracellular bacterium covered by the plasma membrane. (**B**) TEM image showing two cellular compartments in which intracellular bacteria reside. Upper enlarged box shows a bacterium inside a phagosome and the lower enlarged box shows a bacterium in a different compartment situated at the uropod. (**C**) DyLight NHS ester-stained *N meningitidis* FAM20 were allowed to adhere to the uropod of freshly isolated PMNs. Cells were stained with fluorescent labeled Lysotracker. An image of an infected PMN (phase contrast), DyLight NHS ester-stained intracellular meningococci (green), and acidic compartments (red) is shown. Arrows indicate bacteria.

While imaging bacterial adherence to the PMNs by SEM, electrographs frequently showed intracellular diplococci at the uropod, clearly visible underneath the thin layer of the uropod plasma membrane (Fig. 7A). These findings led us to further investigate the possibilities of bacteria entering the PMNs. TEM electrographs showed bacteria in membrane enclosed compartments both at the pseudopod and at the uropod (Fig. 7B). However, the bacteria at the uropod appeared to be in a visually discernible compartment, compared to the phagosomal compartments at the pseudopod (Fig. 7B, close up). To further investigate this in living cells, DyLight 488 NHS-stained bacteria were incubated with freshly isolated PMNs stained with Lysotracker only as contrast (and not for identifying lysosomes). Bacteria were taken up by the uropod (Fig 7C, Movie S3) although phagocytosis by polarized cells is primarily attributed to the pseudopod.

Therefore, these experiments suggest that bacteria can enter the PMN in two different ways: (1) by engulfment/phagocytosis or (2) by bacteria-promoted internalization.

### PMNs transport bound bacteria across epithelial cell layers

**Figure 8:**
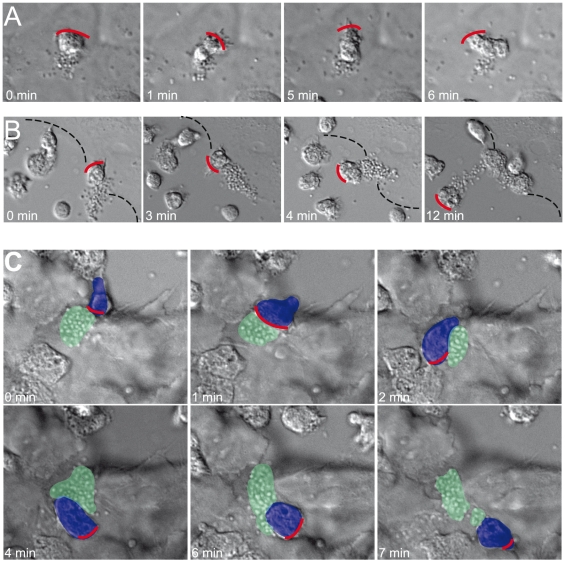
PMN interacts with bacteria bound to epithelial cells. *N. meningitidis* FAM20 were allowed to form microcolonies and adhere to FaDu cells. After one hour, freshly isolated PMNs were added. Representative DIC image sequences at 63x magnification from at least three independent live-cell time-lapse experiments are shown. Red lines indicate the pseudopods and the front of the migrating PMN. (**A**) PMN migrating towards microcolonies but is immobilized and trapped by a microcolony that is bound to both an epithelial cell and a uropod. (**B**) The PMN removes the microcolony from the epithelial cell and transports it away from the site of initial adherence. The dotted black line indicates the borderline of the FaDu cells. (**C**) A microcolony (green) adheres to the PMN (blue) and penetrates a cell layer by hitchhiking on a PMN. The red lines indicate PMN pseudopods and the front of the migrating PMN. Images were processed by Adobe Photoshop.

*N. meningitidis* adheres to the human pharyngeal epithelium. Pharyngeal cells release cytokines that recruit PMNs to the site of infection. *N. meningitidis* FAM20 were allowed to form microcolonies and adhere to epithelial pharyngeal FaDu cells (ATCC HTB-43, LGC Standards). After 90 minutes of infection, freshly isolated PMNs were added to the cell culture medium. PMNs migrated towards microcolonies and although initial contact was made with the pseudopod, the microcolony ended up bound to the uropod. After this, the PMN was either immobilized and trapped by the microcolony that bound both to the epithelial cell and the uropod (Fig 8A, Movie S4) or the PMN was able to remove the microcolony from the epithelial cell and transport it away from the site (Fig 8B, Movie S5). The nature of PMNs allows them to easily migrate under and in between layers of epithelial cells. PMNs with microcolonies attached at the uropod were seen to migrate in between and under a tightly confluent layer of FaDu cells, potentially disrupting the junctions between them (Fig 8C, Movie S6).

These data show how bacteria can use the uropod for transport and thereby spread over an epithelial cell surface with the help of PMNs *in vitro*, suggesting a mechanism that bacteria may utilize during an infection. This observation demonstrates a new route for *Neisseria* to penetrate epithelial layers.

## Discussion

**Figure 9:**
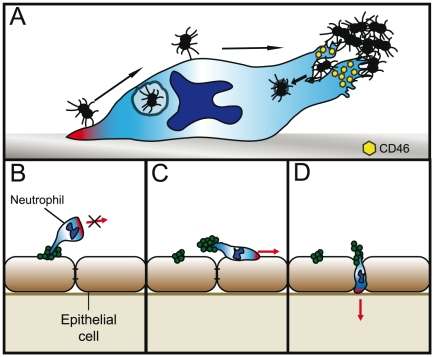
A schematic illustration of bacteria hitchhiking on PMNs. (**A**) Bacterial adherence to PMNs occurs either by direct uropod contact or by transport on the plasma membrane from the pseudopod. Although initial contact was made with the pseudopod, PMNs ended up bound to the microcolony via their uropod. The binding recruits CD46 and bacteria can be internalized via the uropod. (**B**) The PMN can be immobilized and trapped by a microcolony that is bound to both an epithelial cell and a uropod or (**C**) the PMN can remove the microcolony from the epithelial cell and transport it away from the site. (**D**) The nature of PMNs allows them to easily migrate under and in between epithelial cells. In this manner, bacteria can hitchhike on the uropod to penetrate the epithelial barrier.

Recruitment of PMNs from the blood is an early event during bacterial infection. Upon bacterial adherence, host target cells release cytokines that promote PMN infiltration into infected tissue. Pathogenic *Neisseria* encounters high numbers of PMNs during colonization and close contact is inevitable between these cells. Bacterial pathogens often manipulate the immune system to their advantage [44], [45] and *N. gonorrhoeae* is particularly specialized in evading killing by PMNs. Bacterial adherence to the uropod of actively migrating PMNs could be an additional mechanism for bacterial spread and immune evasion. A schematic illustration of the events is presented in Figure 9A.

In this study, we present a novel adherence mechanism for pathogenic *Neisseria*, namely pilus-mediated adherence to the uropod of PMNs. Freshly isolated PMNs from human blood were incubated with bacteria and the interaction was characterized and visualized by live-cell microscopy, TIRF, TEM and SEM. The direct pilus-PMN interaction was clearly shown in both live-cell conditions and high-resolution imaging. Previous studies have yielded contradictory results regarding the role of pili in adhesion and phagocytosis [46], [47]. However, we observed that when a piliated bacterium encountered a pseudopod, it was transported on the plasma membrane to the uropod, thereby evading phagocytosis (Fig. 9A), a phenotype not seen in non-piliated strains. Although some bacteria evaded phagocytosis in this way, we also noticed that the same cell could phagocytose other piliated bacteria. It has previously been shown that protein-coated latex beads can be transported from the pseudopod to the uropod in a similar manner [48], [49].

The adherence to PMNs is more frequent for gonococci than meningococci, corresponding to what is seen with epithelial cells. No adherence was detected for *E. coli*, *S. pyogenes* or type IV-expressing *P. aeruginosa*, suggesting that uropod adherence is specific for *Neisseria.* High levels of adherence were exclusive for pathogenic *Neisseria*, which implies that the uropod adherence is important during infection.

Piliated uropathogenic *E. coli* (UPEC) has been shown to bind uniformly to the surface of PMNs. The adhesion is indirectly mediated by type I pili that leads to CD55 receptor binding [50]. CD55 shows high structural similarity and functionality with CD46 and both glycoproteins are regulators of complement activation. We show that CD46 accumulated at the uropod in close proximity to the adhering *Neisseria*. CD46 does not have a direct role in bacterial adherence to the uropod. However, the role of CD46 in signaling should be investigated further. It is possible that the recruitment of CD46 may function in cellular signaling prior to uptake via the uropod or regulate the production of nitric oxide and interferon as seen in macrophages [51].

In *N. meningitidis*, expression of both PilC1 and PilC2 were required for adherence, indicating that PilC1 and PilC2 may have two different functions, either indirectly in the biogenesis of the pilus filament or directly binding to PMNs *in vitro*. Specific adhesion-promoting regions in PilC1 are most likely involved in this adherence as previously reported [52]. It is possible that PilC1 mediates the adherence as seen in epithelial cells, while PilC2 is involved in the CD46 signaling observed here. This is not due to variance in piliation since inactivation of either *pilC1* or *pilC2* results in the same number of pili [53]. PilC2 exhibits defined cell and tissue tropism [54] and correlates with the CD46-independent binding of PilC2-expressing T4P seen in epithelial cells [55]. Gonococcal adherence occurred in both PilC1-expressing and PilC2-expressing isogenic mutants, showing no differences between PilC1 and PilC2 for uropod adherence.

Intracellular bacteria were often present inside the uropod. Since pilus-mediated adherence of pathogenic *Neisseria* to epithelial cells is followed by invasion, we investigated the possibility that bacteria also invade PMNs. Although it is clearly shown here that bacteria can be internalized in some way via the uropod, it is difficult to separate the number of intracellular bacteria that have entered the phagosome via the pseudopod from those that have entered via the uropod since there is always an unknown time factor for the maturation of the phagolysosome. Bacterial entry via the uropod could be a strategy for pathogenic *Neisseria* to impair phagosomal maturation, thus leading to intracellular survival.

The observed interactions between bacteria and PMNs at the epithelial cell layer revealed the impact of bacterial adhesion to the uropod other than phagocytosis evasion. Addition of PMNs to epithelial cells and bacterial microcolonies impaired the PMNs' ability to migrate and phagocytose bacteria (Fig. 9B). The immobilization was transient and PMNs adhering to the microcolonies already bound to epithelial cells could break away from the epithelial cells and migrate with the attached bacteria away from the site of adhesion, therefore spreading bacteria to uninfected cells. It is possible that the removal of the bacteria by the PMN requires the cooperative action of several PMNs and that it depends on PMN load in the assay (Fig. 9C). Also, PMNs could disrupt cell-to-cell contact and transport bacteria in between the cells, hence potentially giving the bacteria access to the sub-epithelium (Fig. 9D). Several mechanisms have been described that explain how *Neisseria* cross the epithelium: through damaged epithelial layers; the transcellular route; depletion of junctional proteins followed by an opening at the cell-cell interface; and via a “Trojan horse” manner inside phagocytes [56], [57], [58], [59]. Furthermore, the influx of leukocytes at the BBB [60] during meningococcal infections may be a possible way of hitchhiking bacteria to reach the CSF. It is generally assumed that pilus expression plays a major role in the crossing of the BBB by bacteria and is in agreement with data showing the importance of PilC-expressing bacteria for crossing the BBB [61].

In summary, pathogenic *Neisseria* hitchhike on the uropod and thereby utilizing PMNs for immune evasion and dissemination.

## Materials and Methods

### Ethics Statement

N/A.

### PMN isolation and cell lines

Non-coded blood from donors was purchased from the Blood Center and used within 2 hours after collection. Viable PMNs were isolated as described elsewhere using Lympholyte®-poly (Biosite) [62]. The isolation method yielded pure PMNs that were >95% viable as measured by trypan blue staining. Freshly isolated PMNs were maintained in Dulbecco's modified eagle medium (DMEM, Invitrogen) supplemented with 20% inactivated fetal bovine serum (FBS, Gibco) and were always used within 4 hours after isolation. The epithelial pharyngeal cell line FaDu (ATCC HTB-43, LGC Standards) was maintained in DMEM supplemented with 10% inactivated FBS at 37°C and 5% CO_2_.

### Bacterial strains

Strains used in this study are listed in table 2. Bacteria were grown on GCB (GC medium base Difco, Detroit, MI, USA) agar plates containing Kellogg's supplement [4] at 37°C and 5% CO_2_. Piliated (P^+^), translucent phenotypes of gonococcal strains were distinguished by colony morphology under binocular light microscope and re-streaked every 18 to 20 hours to ensure a P^+^, Opa^-^ phenotype. *N. meningitidis* wild-type strain FAM20 belongs to serogroup C and is P^+^ [63]. All bacterial strains determined to be P^+^ have previously been analyzed to express T4P, as determined by electron microscopy and the ability to form microcolonies in solution (data not shown). After adherence assays, bacteria were harvested from the GC agar plate and re-suspended in DMEM. Optical density was measured at 600 nm to calculate the number of bacteria per milliliter.

**Table 2:** Bacterial strains and mutants used in this study.

	Relevant phenotype		
Strain	Pili	PilC1/PilC2	Reference
*N. gonorrhoeae* MS11	yes	PilC+	(Swanson *et al*., 1987)
*N. gonorrhoeae* MS11P-n	no	PilC+	(Swanson *et al*., 1987)
*N. gonorrhoeae* MS11 *ΔpilC1*	yes	PilC2+	(Jonsson *et al.*, 1991)
*N. gonorrhoeae* MS11 *ΔpilC2*	yes	PilC1+	(Jonsson *et al.*, 1991)
*N. meningitidis* FAM20	yes	PilC+	(Moore *et al*., 1995)
*N. meningitidis* FAM20 *ΔpilC1*	yes	PilC2+	(Rahman *et al*., 1997)
*N. meningitidis* FAM20 *ΔpilC2*	yes	PilC1+	(Rahman *et al*., 1997)
*N. meningitidis* FAM20 *ΔpilT*	yes	nd	(Jones *et al*., 2009)
*N. subflava*	yes*	no	this study, (Jonsson *et al*., 1991)
*N. lactamica*	yes*	no	this study, (Jonsson *et al*., 1991)
*S. pyogenes* S165	nd	nd	(Lovkvist *et al*., 2008)
*E. coli* DH5α	nd	nd	
*P. aeruginosa* PA32	yes	nd	

Nd (not determined), * Pili was observed in few bacteria.

### Live cell imaging

Freshly isolated 2.5×10^5^ PMNs in DMEM (20% FBS) were added to glass bottom 12 well dishes (MatTek Corp.). The PMNs were incubated for 30 min at 37°C and 5% CO_2_, allowing the cells to adhere to the glass bottom. The cell culture dishes were transferred to a humidified incubation chamber (37°C, 5% CO_2_) connected to an inverted fluorescence microscope (Cell observer, Carl Zeiss). Upon assay, 2.5×10^7^ bacteria were carefully added to each of the wells. The interaction was monitored in differential interference contrast (DIC) light for 0 to 3 hours. Images of randomly selected fields of vision were captured at different time points. Live-cell time-lapse movies were generated by capturing images of the cells every 5 seconds for 10 minutes. Images and movies were further processed by ImageJ software (National Institutes of Health) and Adobe Photoshop software (Adobe). No differences between bacterial adherence, PMN motility or phagocytic activity could be detected when cells were grown on poly-D-lysine-coated glass or serum-coated glass.

### Transmission and scanning electron microscopy

For SEM, freshly isolated 2.5×10^5^ PMNs in DMEM (20% FBS) were first allowed to adhere to 13 mm uncoated cover glasses (nr. 0, Mentzel) and were then incubated with 2.5×10^7^ *N. meningitidis* FAM20 P^+^ for 3 hours. Cells were fixed in 2.5% glutaraldehyde (Sigma) for 30 minutes and dehydrated by increasing concentrations of ethanol (30%, 50%, 70%, 90% and finally three times in 100% ethanol). The glasses were immersed in 100% hexamethyldisilazane (HMDS, Sigma) for 3 minutes and then dried in a moist free environment. The samples were coated with gold using a sputter coater and examined using a JEOL JSM-7000F.

For TEM, PMNs were incubated with *N. gonorrhoeae* MS11 P^+^ (m.o.i. 100) for 3 hours at 37°C 5% CO_2_. The cells were washed three times with PBS (Accugene) and fixed in 3.7% paraformaldehyde (PFA, diluted in PBS) (Sigma). Cells were then rinsed in 0.1 M phosphate buffer (PB) and transferred to 2.5% glutaraldehyde (Sigma) in 0.1 M PB and stored in a refrigerator. Cells were then post-fixed in 2% osmium tetroxide in 0.1 M PB, pH 7.4, at 4°C for 2 hours, dehydrated in ethanol followed by acetone and embedded in LX-112 (Ladd, Burlington, Vermont, USA). Sections were contrasted with uranyl acetate followed by lead citrate and examined in a Tecnai 12 Spirit Bio TWIN transmission electron microscope (Fei, The Netherlands) at 100 kV. Digital images were taken by a Veleta camera (Olympus Soft Imaging Solutions, GmbH, Münster, Germany)[64].

### Antibodies and immunofluorescence staining

Freshly isolated 5×10^5^ PMNs in DMEM (20% FBS) were added to serum coated glass and infected (MOI 100). The slides were incubated for 2–3 hours at 37°C and 5% CO_2_, allowing the bacteria to interact with the cells. PMNs and bacteria were fixed for 10 minutes in 3.7% PFA in PBS. Cells were permeabilized with 0.2% Triton X-100 for 10 minutes and blocked with Clear back® (MBL international) for 60 minutes at room temperature. Primary antibodies raised against the following targets were used: CD46 (H-294, Santa Cruz 1/50 dilution), CD11b (ICRF44, BioLegend, 1/50 dilution) and CD29 (301302, BioLegend, 1/50 dilution). Goat-anti-rabbit IgG and goat-anti-mouse IgG antibodies conjugated to Alexa fluor 488 nm or Alexa fluor 633 nm (Molecular Probes) were used at a dilution of 1∶500. Coverslips of fixed PMNs and bacteria were mounted in Vectashield containing 4′,6-diamidino-2-phenylindole dihydrochloride (DAPI, Vector laboratories). Lysotracker (75 nM final concentration) was used according to the manufacturer's recommendations (Molecular Probes).

### TIRF microscopy

Freshly isolated 2.5×10^5^ PMNs in DMEM (20% FBS) were allowed to adhere to 35 mm poly-d-lysine-coated glass bottom dishes (MatTek corp.) for 30 minutes. One bacterial colony was re-suspended in 30 µl of sterile PBS. To visualize bacterial proteins, whole viable bacteria were stained with the DyLight 488 NHS ester (Thermo Scientific). The DyLight staining did not affect any bacterial properties. DyLight 488 NHS was added to the bacterial suspension for 5 to 10 minutes at 37°C and 5% CO_2_ (700 µg/ml in PBS). NHS-labeled bacteria were then added to the PMNs and observed under the microscope. Visualization of the pilus interaction with PMNs during live-cell time-lapse analysis was performed with total internal reflection fluorescence (TIRF) microscopy using a connected argon laser and a 100× objective (N/A 1.46, Carl Zeiss).

### Adherence assay to FaDu cells

FaDu cells were cultured in DMEM (10% FBS) on 35 mm poly-d-lysine-coated glass bottom dishes to 60 to 80% cell confluence. During the assay, *N. meningitidis* FAM20 P^+^ was incubated with the FaDu cells (m.o.i 100) for 90 minutes. Unbound bacteria were then carefully washed away and freshly isolated PMNs (2.5×10^5^) were added to the infected FaDu cells. The interaction between the infected cells and PMNs was monitored by DIC light microscopy over time.

### Statistical analysis

Student's *t*-test was used for PMN velocity analyses and data were considered significant if *P*<0.05. A one-way ANOVA was performed in bacterial adherence assays. Data were considered significant if *P*<0.05.

## Supporting Information

Methods S1 **Supporting material and methods.**
(DOC)

Figure S1 **Bacterial adherence to polarized HL-60 cells.** Uropod-specific adherence of *N. meningitidis* FAM20 in differentiated and activated HL-60 cells. Bacteria adhered to the uropod of HL-60 cells. Red lines indicate pseudopods and the front of the migrating PMN. Arrow indicates bacteria.
(EPS)

Figure S2 ***N. subflava*** **and** ***N. lactamica*** **express pili.** Image shows DyLight 488 NHS ester-labeled live bacteria in solution. Pili were visualized in TIRF through a 100 x magnification objective (N/A 1.46). *N. subflava* (left) and *N. lactamica* (right) are shown. Arrows indicate pili.
(EPS)

Movie S1 **Bacteria escape engulfment by the PMN and move along the plasma membrane to the uropod.** Movie shows the interaction between a bacterial microcolony of *N. meningitidis* FAM20 and a PMN. DIC images were captured at 0.2 Hz through a 63 x oil objective. The image sequence was further processed into a movie by ImageJ software.
(AVI)

Movie S2 **Pili directly interact with the uropod.** DyLight 488 NHS-stained *N. meningitidis* FAM20 were incubated with freshly isolated PMNs in glass bottom dishes. Interaction between the pili and PMNs was observed under the microscope in TIRF using a connected argon laser and a 100x oil objective (N/A 1.46). Fluorescence images were captured at 80 Hz. The image sequence was further processed into a movie by ImageJ software.
(AVI)

Movie S3 **Bacteria are internalized at the uropod.** DyLight 488 NHS-stained *N. meningitidis* FAM20 (green) were incubated with freshly isolated Lysotracker-stained (red) PMNs in glass bottom dishes. Cellular DNA was stained with Hoechst 33342 (blue). Bacterial adhesion at the uropod of the activated PMN is followed by internalization of the bacteria. Fluorescence images were captured at 0.1 Hz through a 100x oil objective. The image sequence was further processed into a movie by ImageJ software.
(AVI)

Movie S4 **A PMN is trapped and immobilized by a bacterial microcolony.** *N. meningitidis* FAM20 were allowed to form microcolonies and adhere to FaDu cells. After one hour, freshly isolated PMNs were added. Movie shows a bacterial microcolony bound to both an epithelial cell and a PMN. DIC images were captured at 0.2 Hz through a 63 x objective. The image sequence was further processed into a movie by ImageJ software.
(AVI)

Movie S5 **PMN interacts with bacteria adhering to epithelial cells.** *N. meningitidis* FAM20 were allowed to form microcolonies and adhere to FaDu cells. After one hour, freshly isolated PMNs were added. Movie shows a PMN removing a microcolony from the epithelial cell and transporting it away from the site of initial adherence to FaDu cells. DIC images were captured at 0.2 Hz through a 63 x objective. The image sequence was further processed into a movie by ImageJ software.
(AVI)

Movie S6 **A PMN transports bacteria in between a cell monolayer.** *N. meningitidis* FAM20 were allowed to adhere to FaDu cells. After one hour of infection, freshly isolated PMNs were added to the cell culture medium. Movie shows a microcolony adhering to a PMN. The PMN penetrates the layer of cells, transporting the bacteria between the cells. DIC images were captured at 0.2 Hz through a 63 x objective. The image sequence was further processed into a movie by ImageJ software.
(AVI)
