# Supplementary material for: Pathogenic Neisseria Hitchhike on the Uropod of Human Neutrophils
Source: PLoS One. 2011 Sep 16;6(9):e24353. doi: 10.1371/journal.pone.0024353 (PMC3174955; doi:10.1371/journal.pone.0024353)
Supplement: Methods S1 — Supporting material and methods. (DOC) [file pone.0024353.s001.doc]

*Growth of HL-60 cells and differentiation*

HL-60 cells (ATCC CCL-240) was maintained in DMEM supplemented with 20% inactivated fetal bovine serum at 37°C, 5% CO2. HL-60 cells were differentiated to PMN-like cells by the addition of 1.3% DMSO for 5 days prior assay. HL-60 cells were further activated with the addition of 1µM N-formyl-MET-LEU-PHE (fMLP, Sigma, Saint Louis, USA) for 30 min prior bacterial adhesion assay.

*TIRF microscopy of commensal* Neisseria

One 10 μl loop of bacteria was resuspended in 100 μl PBS and stained for 5-10 minutes at 37°C with DyLight 488 NHS ester (700 μg/ml, Thermo Scientific). Stained bacteria were added to poly-d-lysine coated glass-bottom dishes (MatTek corp.) containing pre warmed GC broth (15 g Protease Peptone no. 3,1 g soluble starch, 4 g K_2_HPO_4_, 1 g KH_2_PO_4_, 5 g NaCl to 1 L). The dishes were centrifuged for 7 min at 200 x g to sediment bacteria close to the glass surface. Visualization of the pilus interaction with PMNs during live-cell time-lapse analysis was performed with total internal reflection fluorescence (TIRF) microscopy using a connected argon laser and a 100 x objective (N/A 1.46, Carl Zeiss).
